# The Roles Played by Long Non-Coding RNAs in Glioma Resistance

**DOI:** 10.3390/ijms22136834

**Published:** 2021-06-25

**Authors:** Yeonsoo Chae, Jungwook Roh, Wanyeon Kim

**Affiliations:** 1 Department of Science Education, Korea National University of Education, Cheongju-si 28173, Chungbuk, Korea; yeonss012@gmail.com (Y.C.); junguk1112@knue.ac.kr (J.R.); 2 Department of Biology Education, Korea National University of Education, Cheongju-si 28173, Chungbuk, Korea

**Keywords:** chemotherapy, glioma, immunotherapy, long non-coding RNA, radiotherapy, therapy resistance

## Abstract

Glioma originates in the central nervous system and is classified based on both histological features and molecular genetic characteristics. Long non-coding RNAs (lncRNAs) are longer than 200 nucleotides and are known to regulate tumorigenesis and tumor progression, and even confer therapeutic resistance to glioma cells. Since oncogenic lncRNAs have been frequently upregulated to promote cell proliferation, migration, and invasion in glioma cells, while tumor-suppressive lncRNAs responsible for the inhibition of apoptosis and decrease in therapeutic sensitivity in glioma cells have been generally downregulated, the dysregulation of lncRNAs affects many features of glioma patients, and the expression profiles associated with these lncRNAs are needed to diagnose the disease stage and to determine suitable therapeutic strategies. Accumulating studies show that the orchestrations of oncogenic lncRNAs and tumor-suppressive lncRNAs in glioma cells result in signaling pathways that influence the pathogenesis and progression of glioma. Furthermore, several lncRNAs are related to the regulation of therapeutic sensitivity in existing anticancer therapies, including radiotherapy, chemotherapy and immunotherapy. Consequently, we undertook this review to improve the understanding of signaling pathways influenced by lncRNAs in glioma and how lncRNAs affect therapeutic resistance.

## 1. Introduction

Gliomas include several types of tumors originated from non-neoplastic glial cells in the central nervous system. Gliomas are traditionally classified into four grades based on specific features of the tumor, such as proliferative and differential potential, invasiveness, nuclear dysplasia, mitotic activity, necrosis and vascular hyperplasia [1]; however, accumulated studies have shown molecular basis of tumorigenesis in gliomas and the World Health Organization (WHO) has updated the classification of gliomas according to molecular characteristics, such as IDH status and 1p/19q codeletion [2,3,4]. High grade gliomas are the most common primary malignant brain tumors in adults and account for approximately 80% of brain tumors. These tumors are the most common and fatal form of cancer in the central nervous system and include anaplastic astrocytoma, anaplastic oligodendroglioma and glioblastoma [5,6]. As they have poorer survival rates than other cancers [7,8,9], it is important to tailor the treatment to individual patients. Research on the molecular profiling of cancer has intensified due to its clinical value for the diagnosis, prognosis, and treatment of gliomas. Complex genetic profile analysis of glioma has revealed alterations in the genes responsible for several signaling pathways, including the p53 pathway, the receptor tyrosine kinase pathway, phosphoinositide 3-kinase (PI3K) pathway and the retinoblastoma (Rb) pathway [10,11], and these alterations circumvent cell cycle checkpoints, aging and apoptosis pathways and lead to a failure to regulate cell death and in so doing enhance cell viability [12]. Various pieces of evidence have emerged to suggest that lncRNAs cause cancer by altering these signaling pathways [13].

Approximately 98% of RNA in human DNA is not translated into protein, and thus, is called non-coding RNA (ncRNA) [14]. These RNAs, which are classified as microRNAs (miRNAs) or long non-coding RNAs (lncRNAs), interfere with the transcription and translation of genes without altering DNA sequences [15]. lncRNAs are longer than 200 nucleotides; dysfunctions of several lncRNAs have often been reported in different tumors; and these dysfunctional lncRNAs are associated with the pathogenesis and regulation of glioma development, including cell proliferation, cell motility, angiogenesis, drug resistance and radiation resistance [16,17,18]. Furthermore, the expression profiles of abnormal lncRNAs in clinical glioma specimens have been shown to be correlated with malignant grade and histological differentiation, which has important clinical implications for glioma diagnosis and prognosis [19,20]. For example, the expression of homeobox A transcript antisense RNA myeloid-specific 1 (HOTAIRM1) is positively correlated with WHO grade as well as histopathologic classification according to both The Cancer Genome Atlas (TCGA) and Chinese Glioma Genome Atlas (CGGA) sets [21]. In the results of the genetic analysis of glioma patients, the dysregulation of functional lncRNAs was detected. These expression levels of lncRNAs derived from blood or cerebrospinal fluid as well as surgical tissues were clinically related to glioma diagnosis in several liquid biopsy studies [22,23]. On the one hand, lncRNAs that are sensitive to radiation therapy, chemotherapy and immunotherapy are also being discovered [24,25,26]. Studies have shown that some lncRNAs such as HOTAIRM1 and colon cancer-associated transcript 2 (CCAT2), associated with tumorigenesis and cancer development, were upregulated in glioma, while other lncRNAs showing tumor-suppressive functions, such as RP11-838N2.4 and MALAT1, were downregulated. From studies on the relationship between these lncRNAs and therapeutic resistance, it would be expected that the efficient therapies can be achieved in glioma patients. Thus, the investigation of lncRNAs and their signaling pathways at the molecular level provides a great opportunity for developing novel therapeutic strategies. This review addresses the functions of lncRNAs in glioma and provides an overview of the association between lncRNAs and glioma therapeutic resistance.

## 2. Dysregulated lncRNA in Glioma

lncRNAs can play crucial roles in various signaling pathways, including cell growth, apoptosis and differentiation, by acting as a sponge for miRNAs, and thus regulating mRNA expression [27,28,29,30,31]. RNAs competing with miRNA are defined as competing endogenous RNAs (ceRNAs), and several lncRNAs are described as ceRNAs affecting intracellular signaling pathways [32]. According to previous studies, dysregulations of lncRNAs are critical in the pathogenesis, progression and malignancy of glioma [33,34]. It was well established that the activation of the PI3K/Akt signaling pathway is highly responsible for the facilitation of glioma tumor formation. lncRNA brain cytoplasmic RNA 1 (BCYRN1) acts as a ceRNA that suppresses glioma progression accompanied by the inhibition of Akt signaling [35]. BCYRN1 directly binds to and sponges miR-619-5p, leading to the upregulation of the CUE domain containing protein 2 (CUEDC2), phosphatase and tensin homolog deleted on chromosome ten (PTEN) and p21 with the inactivation of Akt. In addition, lncRNA X-inactive specific transcript (XIST) can be associated with activation of the PI3K/Akt signaling by sponging miR-126, leading to a glucose metabolic switch, cell proliferation, epithelial–mesenchymal transition (EMT) induction and apoptosis resistance, which contribute to glioma progression and malignancy [36]. Profiling analysis results have confirmed that patients suffering from glioma exhibit different lncRNA expression patterns [33], and thus, lncRNAs have attracted considerable attention as potential biomarkers of glioma diagnosis, prognosis and targeted therapy [37]. From this point of view, the identification of glioma-specific lncRNAs is required for diagnosing disease stage and identifying optimal treatment strategies. 

### 2.1. Upregulations of Oncogenic lncRNAs in Glioma

Several lncRNAs that function as oncogenes in tumor cells have been identified, and it has been shown that some oncogenic lncRNAs are upregulated in glioma cells (Table 1). lncRNA Hox transcript antisense intergenic RNA (HOTAIR) is involved in cell cycle progression in U87 and LN229 glioma cell lines and intracranial xenograft tumors of nude mice due to their interaction with enhancer of zeste homolog 2 (EZH2) [38] and also acts as a ceRNA that promotes tumor progression by sponging miRNA responsible for producing tumor microenvironments [33]. The elevated expression of HOTAIRM1 is positively associated with cell proliferative ability, EMT induction, and chemoresistance in glioma cells and with poor overall survival rates in glioma patients [21,39]. Overexpressed lncRNA CCAT2 in human glioma cell lines might be released in an exosome-dependent manner, and this release leads to the suppression of endothelial cell apoptosis via the downregulation of Bax and caspase 3, which are responsible for angiogenesis induction, glioma development and chemoresistance [40,41]. lncRNA colorectal neoplasia differentially expressed (CRNDE) promotes the proliferation and invasion of U87 and U251 glioma cell lines and patient-derived glioma tissues by acting as a ceRNA and sponging miR-136-5p, which has been linked with poor prognosis in glioma patients [19,42]. In addition, lncRNA H19 induces the proliferation and EMT of several glioma cell lines and human glioma tissues by sponging miRNAs including miR-152 and miR-130a-3p [43,44]. lncRNA plasmacytoma variant translocation 1 (PVT1) is upregulated in human glioma tissues and cell lines and functions as a miRNA sponge, binding to miR-128-3p [45]. The overexpression of PVT1 and downregulation of miR-128-3p provoke the upregulation of gremlin 1 (GREM1), promoting cell proliferation, invasion and migration whilst inhibiting apoptosis in glioma cells. LINC00689 interacts with miR-338-3p, leading to the upregulation of pyruvate kinase M2 (PKM2) associated with the cell growth, metastasis and glycolytic switch of patient-derived glioma tissues and glioma cell lines [46]. As a result, oncogenic lncRNAs tend to be overexpressed in glioma cells, and their upregulation is positively related to the pathogenesis and progression of glioma via the regulation of intracellular tumorigenic responses.

### 2.2. Downregulation of Tumor-Suppressive lncRNAs in Glioma

In addition to oncogenic lncRNAs, some lncRNAs have tumor-suppressive activities in glioma cells (Table 2). lncRNA RP11-838N2.4 is downregulated in glioblastoma cell lines, and this downregulation is associated with temozolomide (TMZ) resistance and poor prognosis in glioblastoma patients [51]. Another lncRNA metastasis-associated lung adenocarcinoma transcript 1 (MALAT1) inhibits cell proliferation and invasion through the downregulation of extracellular signal-regulated kinase (ERK), matrix metalloproteinase 2 (MMP2) and MMP9 in U87 and U251 glioma cells and glioma nude mice models [52]. Moreover, MALAT1 plays a tumor-suppressive role in glioma cells by sponging miR-155, which leads to F-box/WD repeat-containing protein 7 (FBXW7) expression, and its downregulation in glioma tissues is associated with poor survival [53]. Taurine upregulated gene 1 (TUG1) is a lncRNA upregulated by taurine treatment [54], and reportedly, TUG1 is downregulated in glioma tissues; this downregulation is associated with the inhibition of caspase 3/9 activation and the induction of Bcl2, which are considered to be responsible for indicators of poor prognosis in glioma patients [55]. A study performed to identify prognostic lncRNAs using TCGA datasets reported that high expressions of five lncRNAs, that is, prostate androgen-regulated transcript 1 (PART1), MGC21881, myocardial infarction-associated transcript (MIAT), growth arrest specific 5 (GAS5) and Prader Willi/Angelman region RNA 5 (PAR5), were correlated with prolonged survival in glioblastomas [56]. lncRNA PART1 was reported to be significantly downregulated in glioma tissues and cell lines, and in the same study, PART1 promoted apoptosis and inhibited the proliferation of glioma cells by sponging miR-190a-3p, and subsequently upregulating PTEN and inactivating Akt signaling [57]. In addition, lncRNA MIAT upregulation is associated with increased blood–tumor barrier permeability, which is an important requirement for drug delivery to brain tumors, and this increase in blood–tumor barrier permeability was attributed to the sponging of miR-140-3p by MIAT and subsequent activation of ZO-1-associated kinase (ZAK) and nuclear factor kappa B (NF-κB) [58]. Additionally, the upregulation of lncRNA PAR5 resulted in its binding to EZH2 and the inhibition of cell proliferation, invasion, and migration in U87 and U251 human glioma cells; notably, PAR5 expression is positively related to prognosis in glioma patients [59]. As shown in the above reports, tumor-suppressive lncRNAs tend to be downregulated in glioma and inhibit major cellular responses commonly observed in glioma cells.

## 3. lncRNAs Associated with Therapeutic Resistance in Glioma

Radiation therapy, chemotherapy and immunotherapy in addition to surgical resection have been suggested for glioma treatment [63,64,65]. Unfortunately, however, there are cases of patients who are resistant to combinational therapies [66]. As previously stated, several lncRNAs act as miRNA sponges, which are significant features for the regulation of therapeutic resistance in glioma therapy, since miRNAs play crucial roles in intracellular signaling and cell–cell communication for cancer development. Additionally, from this point of view, lncRNAs as well as miRNAs deeply involved in resistance and sensitization to cancer therapies have attracted a large amount of attention as potential diagnostic and prognostic biomarkers for glioma [67,68,69,70]. For example, miR-21 is related to various cellular responses regulating radio- and/or chemosensitivity [71], and miR-301a promotes radiation resistance to glioma cells by downregulating a tumor suppressor gene, transcription elongation factor A-like 7 (TCEAL7) [72]. Accumulating evidence indicates that lncRNAs can interact with these miRNAs and regulate their expressions, which suggests lncRNAs play important roles in the formation of tumor microenvironments and the acquisition of therapeutic resistance [73,74]. lncRNA NCK1 antisense RNA 1 (NCK1-AS1) directly interacts with miR-22-3p, resulting in the depletion of miR-22-3p and promotion of cell proliferation, radioresistance and chemoresistance in glioma [75]. LINC00470 is reported to promote cell proliferation, invasion and TMZ resistance through sponging miR-134 [76]. Thus, studies are required on the molecular networks linking lncRNAs and miRNAs to gain an improved understanding of the mechanism of therapeutic resistance, as this would provide an opportunity to establish therapeutic strategies and improve treatment efficacy for glioma.

### 3.1. Roles of lncRNAs in Glioma Radiotherapy

Radiotherapy is commonly used to treat glioma and is frequently accompanied by TMZ administration after surgical resection [77]. Radiotherapy has evolved, and intensity-modulated radiotherapy, proton beam therapy and radioimmunotherapy have recently been shown to improve treatment efficacy with the alleviation of adverse effects [78]. However, radioresistant glioma patients face the possibility of experiencing adverse effects, especially after adjustments of irradiation strategies [79,80]. Thus, the assessment of radiosensitivity would make it possible to increase treatment efficacy and mitigate harmful adverse effects.

lncRNAs affect radioresistance by regulating various cellular responses, including DNA damage, apoptosis, EMT and several signaling pathways [81]. For example, lncRNA XIST inhibits the expression of miR-329-3p, and miR-329-3p downregulation provokes cell proliferation and invasiveness and the upregulation of cAMP responsive element binding protein 1 (CREB1) expression, thus inducing radioresistance in glioma patients [18]. The expression of lncRNA HLA complex P5 (HCP5) was positively correlated with glioma malignancy and HCP5 knockdown promoted cellular senescence and sensitivity to radiation and inhibited cell proliferation by sponging miR-128 [82]. lncRNA transmembrane phosphatase with tensin homology pseudogene 1 (TPTEP1) has been demonstrated to act as a tumor suppressor in glioma cells, and the upregulation of TPTEP1 in glioma patients has shown an improvement in overall survival probability [60]. TPTEP1 attenuates miR-106a-5p, and the depletion of miR-106a-5p leads to cell stemness and radioresistance in glioma cells [83]. A meta-analysis of 167 low-grade glioma patients after radiotherapy found that 8 lncRNAs (LINC01447, AC004832.1, AC020659.1, AC087241.4, AC092343.1, AL157831.2, DISC1FP1 and FAM30A) were highly correlated with the regulation of radiosensitivity in an mRNA-dependent manner [79]. In addition, several lncRNAs were identified as prognostic candidates of overall survival (LINC01447, AC023796.1, AC000061.1, AL078605.1, LINC01163, LINC02237, AC073324.2, AC023905.1, AL133415.1 and AC106786.1) or progression-free survival (LINC01447, LINC02237, AC106786.1, KC6, GS1-24F4.2, LINC01163, AC000061.1, AL133415.1, AL137005.1 and AC046168.2) after radiotherapy in low-grade glioma patients. Furthermore, the authors suggested that in low-grade glioma, AL133415.1, LINC01447 and AC106786.1 are associated with poor prognoses but that AC000061.1, LINC01163 and LINC02237 are associated with good prognoses for radiotherapy in low-grade glioma. 

Circular RNAs (circRNAs) are another class of non-coding RNAs and reportedly play crucial roles in gene regulation by acting as ceRNAs and sponging miRNAs such as lncRNAs [84,85]. In a recent study, altered circRNA expression patterns were observed in glioma cells; these included 10 upregulated (circATP8B4, circCCDC134, circMARCH6, circLRRK1, circANAPC1, circNSD2, circUBAP2, circSEPT9 and circTBC1D1) and 10 downregulated (circRNF216, circMTCL1, circNR2C1, circATAD2, circCSPP1, circUBQLN1, circZDHHC21, circDLG1, circOSBPL10 and circSNX2) circRNAs [47]. It was suggested that circATP8B4 promotes radioresistance by sponging miR-766-5p in glioma cells and that circATP8B4 in extracellular vesicles, such as exosomes released by radioresistant glioma cells, contributes to the acquisition of radioresistant properties by glioma cells.

The experimental results demonstrated that many lncRNAs participate in the regulation of radioresistance, which is intricately connected to irradiation-induced cell death. These results indicate that profiling the expressions of lncRNAs in glioma patients could provide critical information useful for predicting disease progression and for determining a means of increasing the efficacy of radiotherapy.

### 3.2. Roles of lncRNAs in Glioma Chemotherapy

TMZ is a universal DNA-alkylating agent and used as a first-line chemotherapeutic agent in glioma. TMZ induces DNA damage in glioma cells and overwhelms DNA repair system activity, leading to cytotoxicity and apoptosis [86,87,88,89]. However, O^6^-methylguanine DNA methyltransferase (MGMT) is capable of repairing cytotoxic groups and is the prevalent reason for TMZ resistance in glioma patients [90,91,92]. In addition, several molecular mechanisms affecting signaling pathways contributing to the expression and activation of MGMT are highly responsible for the poor outcomes of TMZ-resistant glioma patients [70]. lncRNA FoxD2 adjacent opposite strand RNA 1 (FoxD2-AS1) shows high expression in glioma patients, correlated with poor patient outcomes, especially with drug responses induced by TMZ administration [93]. FoxD2-AS1 inhibits the methylation of the MGMT promoter, thus promoting TMZ resistance [94]. Additionally, temozolomide-associated lncRNA in glioblastoma recurrence (lnc-TALC) is reported to act as a ceRNA for miR-20b-3p and provokes the upregulation of MGMT through regulating the c-Met signaling pathway, resulting in TMZ resistance, tumor development and progression [95].

According to Zhang et al., the expression of lncRNA SET-binding factor 2 antisense RNA 1 (SBF2-AS1) is upregulated in TMZ-resistant glioma tissues as compared with TMZ-sensitive tissues, indicating that SBF2-AS1 is associated with chemoresistance to TMZ [48]. The authors also suggested that exosomal SBF2-AS1 secreted by TMZ-resistant cells might spread TMZ resistance to nearby glioma cells and that SBF2-AS1 might downregulate miR-151a-3p in a ceRNA network manner, upregulate X-ray repair cross-complementing protein 4 (XRCC4) and promote the repair of TMZ-induced DNA damage [49]. Another study showed that the expression of lncRNA urothelial carcinoma associated 1 (UCA1) was upregulated in several glioma cell lines as compared with normal astrocytes, and that UCA1 overexpression increased the IC50 values of cisplatin and TMZ in glioma cell lines, indicating that UCA1 is associated with the promotion of chemoresistance to several antitumor agents [96]. Furthermore, it has been reported that the expression of miR-10a might contribute to the induction of TMZ resistance in glioma cells [97]. In another study, miR-10a was sponged by lncRNA RP11-838N2.4, and the overexpression of RP11-838N2.4 increased TMZ sensitivity in GBM cells [51]. lncRNA AC003092.1 also acts as ceRNA for miR-195, leading to the increased expression of tissue factor pathway inhibitor 2 (TFPI2) [98]. TPFI2 provokes cell apoptosis induced by TMZ treatment; therefore, the overexpression of AC003092.1 inhibits cell proliferation and promotes cell apoptosis, enhancing TMZ sensitivity in glioblastoma.

Recently, lncRNA P73 antisense RNA 1T (TP73-AS1) has attracted attention for reportedly playing a pivotal role in the epigenetic regulation of gene expression in glioblastoma and for its relevance to stemness in glioblastoma cancer stem cells [99]. TP73-AS1 is positively correlated with the metabolisms of nucleosides and proteins, and high levels of TP73-AS1 expression promote TMZ resistance by inducing the expression of aldehyde dehydrogenase 1 family member A1 (ALDH1A1), which is known to maintain stemness and promote chemoresistance in several solid tumors. lncRNA Sex-determining region Y-box 2 (SOX2) overlapping transcript (SOX2OT) is also an epigenetic regulator in glioblastoma and binds to RNA demethylase called AlkB homolog 5 (ALKBH5), which demethylates SOX2 transcripts and results in the expression of oncogenic SOX2, which is deeply related to cell viability and apoptosis [100]. SOX2OT escalates TMZ resistance by inducing the expression of SOX2, which is in line with the poorer overall survival and prognosis of glioblastoma patients expressing high levels of SOX2OT.

As stated above, regulations of miRNA expression by lncRNAs modulate resistance to chemotherapeutic drugs. Selecting proper drug types and titers is critical for chemotherapy, and thus, knowledge of the concentrations of specific lncRNAs would be helpful during treatment planning. Furthermore, establishing strategies targeting those lncRNAs can provide alternative options for TMZ-resistant glioma patients. 

### 3.3. Roles of lncRNAs in Glioma Immunotherapy

Accumulating evidence shows that lncRNAs play fundamental roles in aspects of immune responses such as inflammation, cell differentiation and immune cell maturation and infiltration [101,102,103]. Additionally, some immune-related lncRNAs can be used for cancer type classification as their expressions are correlated with immunologic molecules such as cytokines and chemokines [104,105]. Thus, it is important that immune-related lncRNAs affecting glioma-associated immune cells are identified, as this would aid the development of immunotherapies. In a study that analyzed immune-related lncRNAs across cancer types including breast, lung, colon, skin and brain cancers, immune-related lncRNA levels were highly correlated with immune cell infiltration in many of the examined cancers, and the proportion of immune-related lncRNAs in glioblastoma was higher than that in low-grade glioma [103].

In addition, it was shown that 10 lncRNAs, namely, LINC00944, MIR3142HG, LINC01871, LINC01281, RP11-32F22.2, CTA-384D8.35, LINC01934, LINC00996, CTB-61M7.2 and SMIM25, were responsible for regulating immune pathways. Others have reported that lncRNAs, including the non-coding repressor of the nuclear factor of activated T cells (NRON), long intergenic non-coding RNA for kinase activation (LINK-A), PVT1, MIAT and ITGB2 Antisense RNA 1 (ITGB2-AS1), are involved in cancer-associated immune responses, including T-cell activation, cancer cell antigen presentation and CD8 T-cell infiltration [103,106,107,108]; it has also been reported that the lncRNA MIR155 host gene (MIR155HG) is upregulated in glioblastoma tissues versus their normal counterparts and that its expression is positively correlated with those of immune checkpoint inhibitors, including programmed cell death 1 ligand (PD-L1) and T cell immunoglobulin and mucin domain 3 (TIM-3) in glioblastoma, and related to poor prognosis [50]. According to another study, the expression of lncRNA DiGeorge syndrome critical region gene 5 (DGCR5) was decreased in low-grade glioma and GBM tissues, and DGCR5 is deeply related to the regulation of several immune responses proven by TCGA and CGGA datasets [61,62]. Specifically, DGCR5 showed a negative correlation with immunoregulatory factors, such as programmed cell death 1 (PD-1), PD-L1, LAG3, TIM-3 and B7-H3, and the infiltration capacity of immune cells. In addition, the downregulation of DGCR5 was mainly responsible for the poor prognosis of glioma patients. A recent study reported that, among glioma patients classified according to immunity levels (low, medium or high), those in the high immunity group had the poorest prognosis and lowest survival rate [109]. Five lncRNAs (AP001007.1, LBX-AS1, MIR155HG, MAPT-AS1 and LINC00515) have been suggested to be associated with immune-related genes, including human leukocyte antigen (HLA), PD-L1, TIM-3, B7-H3 and cytotoxic T lymphocyte-associated antigen 4 (CTLA4), and these five lncRNAs were further categorized into risk factor (AP001007.1, LBX-AS1 and MIR155HG) and protective factor (MAPT-AS1 and LINC00515) groups based on their relationships with patient risk, survival time and overall death rate. Moreover, the results of univariate Cox regression and the Least Absolute Shrinkage and Selection Operator (LASSO) regression based on TCGA datasets showed that 11 immune-related lncRNAs, namely, AL391422.4, AC012558.1, AC074286.1, DGCR9, AC126407.1, AL645608.6, FLJ16779, AL355916.1, LINC00641, AC021739.2 and AC124016.2, are associated with the prognosis of low-grade glioma, with 10 of them acting as risk factors and only AL391422.4 acting as a protecting factor [110].

As demonstrated by the abovementioned studies, lncRNAs are receiving a large amount of attention as immunotherapy targets in glioma. Since the expression of immune-related lncRNAs tends to be perturbed in glioma, and the clinical features of glioma patients under immunotherapy are dependent on the expression of lncRNA, accumulating studies on immune-related lncRNAs are valuable for optimizing the efficacy of immunotherapy.

## 4. Clinical Applications of lncRNAs in Glioma

**Figure 1:**
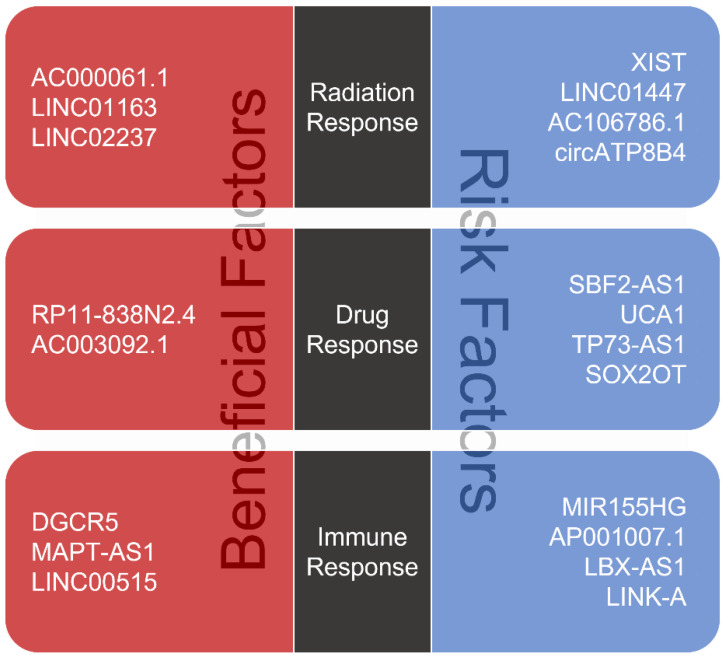
lncRNAs can regulate sensitivity or resistance to different types of anticancer therapies, including radiotherapy, chemotherapy and immunotherapy. lncRNAs associated with increasing therapeutic sensitivity act as beneficial factors and must be upregulated to improve efficacy of glioma treatments. On the other hand, lncRNAs associated with increasing therapeutic resistance act as risk factors and must be downregulated.

As lncRNAs influence the expression of oncogenic and tumor-suppressive miRNAs that regulate intracellular pathways, lncRNAs directly affect resistance or sensitivity to several different types of therapies. Some glioma patients struggle with therapeutic resistance, and their abilities to overcome therapeutic resistance are critical. As lncRNAs such as AC000061.1, RP11-838N2.4 and DGCR5 are known to increase therapeutic sensitivity, they can be defined as beneficial factors in anticancer therapies (Figure 1). On the other hand, lncRNAs including XIST, SBF2-AS1 and MIR155HG, known for increasing therapeutic resistance, can be described as risk factors, and it might be helpful to downregulate these lncRNAs to improve the effects of anticancer therapies. Interestingly, several authors have suggested means of modulating therapeutic resistance using lncRNAs. Applications of RNAi techniques targeting specific lncRNAs offer a potentially effective route forward. For example, glioma cells treated with LINC01447-specific siRNAs or AC106786.1-specific siRNAs exhibited higher apoptosis rates 48 h after irradiation than controls [79]. Knockdown of SOX2OT by SOX2OT-specific short hairpin RNA (shRNA) significantly enhanced TMZ sensitivity, resulting in a decrease in tumor growth in xenograft mice models using glioma cell lines [100], and LINC01116 knockdown mediated by LINC01116-specific shRNA inhibits cell proliferation, migration and invasion in glioma cell lines, and the cultured medium obtained from glioma cells treated with LINC01116-specific shRNA suppressed the ability of tubule formation in human umbilical vein endothelial cells [111]. Additionally, CRISPR systems might be useful to achieve the stable knockdown of lncRNAs. TP73-AS1 depletion in a CRISPR/Cas9 dependent manner attenuated stemness capacity in terms of self-renewal and sphere formation and enhanced the sensitivity of glioblastoma cancer stem cells to TMZ [99]. The upregulation of lncRNAs may also provide a means of enhancing sensitivity to anticancer therapy. RP11-838N2.4 overexpression by transfection caused glioblastoma cells to become highly sensitive to TMZ [51]. Xenograft animal models transplanted with lncRNA AC003092.1-overexpressing glioma cells using the lentiviral system showed a decrease in tumor size after TMZ treatment compared to animals transplanted with negative control cells [98]. Furthermore, exosomes, including lncRNA PTENP1, secreted by human umbilical cord mesenchymal stem cells inhibited glioma cell growth, which indicates that exosomes might be useful for introducing lncRNAs in glioma [112]. Thus, studies have demonstrated that various methods might be employed to adjust the expression of lncRNAs and enhance therapeutic efficacies.

## 5. Conclusions

In this review, we summarize the lncRNAs regulated in glioma and their relationships with different therapies. lncRNAs could be used as promising biomarkers and regulators of signaling pathways associated with therapeutic resistance, and thus are helpful for determining individualized treatment. More importantly, we believe that an improved understanding of the functions and molecular inter-relationships of lncRNAs could lead to promising developments in the glioma treatment field, and further study of molecular mechanisms of therapeutic-resistance-associated lncRNAs is required.

## Figures and Tables

**Table 1:** Oncogenic lncRNAs upregulated in glioma.

lncRNA	Interacting Molecules	Effects of lncRNA in Glioma Cells	Ref.
HOTAIRM1	miR-495-3p and miR-129-5p	Promoting cell proliferation, EMT and TMZ resistance	[21]
CCAT2	-	Suppressing endothelial cell apoptosis leading to angiogenesis	[40]
CRNDE	miR-136-5p	Promoting cell proliferation and invasion	[19,42]
H19	miR-152 and miR-130a-3p	Promoting cell proliferation and EMT	[43,44]
PVT1	miR-128-3p	Promoting cell proliferation, invasion and migration and inhibiting apoptosis	[45]
LINC00689	miR-338-3p	Promoting cell growth, metastasis and glucose metabolism	[46]
XIST	miR-329-3p	Promoting cell proliferation, invasion and inhibiting cell apoptosis	[18]
circATP8B4	miR-766-5p	Promoting cell proliferation and radioresistance	[47]
SBF2-AS1	miR-151a-3p and miR-338-3p	Suppressing cell apoptosis and growth inhibition induced by TMZ, and stimulating cell viability, migration and tube formation of endothelial cells	[48,49]
MIR155HG	-	Promoting cell growth and expression of immune checkpoint inhibitors	[50]

**Table 2:** Tumor-suppressive lncRNAs downregulated in glioma.

lncRNA	Interacting Molecules	Effects of lncRNA in Glioma Cells	Ref.
RP11-838N2.4	miR-10a	Enhancing sensitivity of TMZ	[51]
MALAT1	miR-155	Inhibiting cell viability and proliferation and invasion	[52]
TUG1	caspase3, caspase 9, and BCL-2	Inhibiting cell proliferation and promoting cell apoptosis	[55]
PART1	miR-190a-3p	Inhibiting cell proliferation and promoting cell apoptosis	[57]
MIAT	miR-140-3p	Increasing blood–tumor barrier permeability	[58]
PAR5	EZH2	Inhibiting cell proliferation, invasion and migration	[59]
TPTEP1	miR-106a-5p	Inhibiting cell stemness and radioresistance	[60]
DGCR5	miR-21-3p and miR-23a-5p	Inhibiting Wnt/β-catenin signaing pathway and promoting cell apoptosis, migration and invasion	[61,62]
